# Ebolavirus: Comparison of Survivor Immunology and Animal Models in the Search for a Correlate of Protection

**DOI:** 10.3389/fimmu.2020.599568

**Published:** 2021-02-19

**Authors:** Stephanie Longet, Jack Mellors, Miles W. Carroll, Tom Tipton

**Affiliations:** ^1^ Public Health England, National Infection Service, Salisbury, United Kingdom; ^2^ Nuffield Department of Medicine, University of Oxford, Oxford, United Kingdom

**Keywords:** Ebolavirus, correlate of protection, animal models, survivors, vaccine

## Abstract

Ebola viruses are enveloped, single-stranded RNA viruses belonging to the *Filoviridae* family and can cause Ebola virus disease (EVD), a serious haemorrhagic illness with up to 90% mortality. The disease was first detected in Zaire (currently the Democratic Republic of Congo) in 1976. Since its discovery, Ebola virus has caused sporadic outbreaks in Africa and was responsible for the largest 2013–2016 EVD epidemic in West Africa, which resulted in more than 28,600 cases and over 11,300 deaths. This epidemic strengthened international scientific efforts to contain the virus and develop therapeutics and vaccines. Immunology studies in animal models and survivors, as well as clinical trials have been crucial to understand Ebola virus pathogenesis and host immune responses, which has supported vaccine development. This review discusses the major findings that have emerged from animal models, studies in survivors and vaccine clinical trials and explains how these investigations have helped in the search for a correlate of protection.

## Introduction

**Figure 1:**
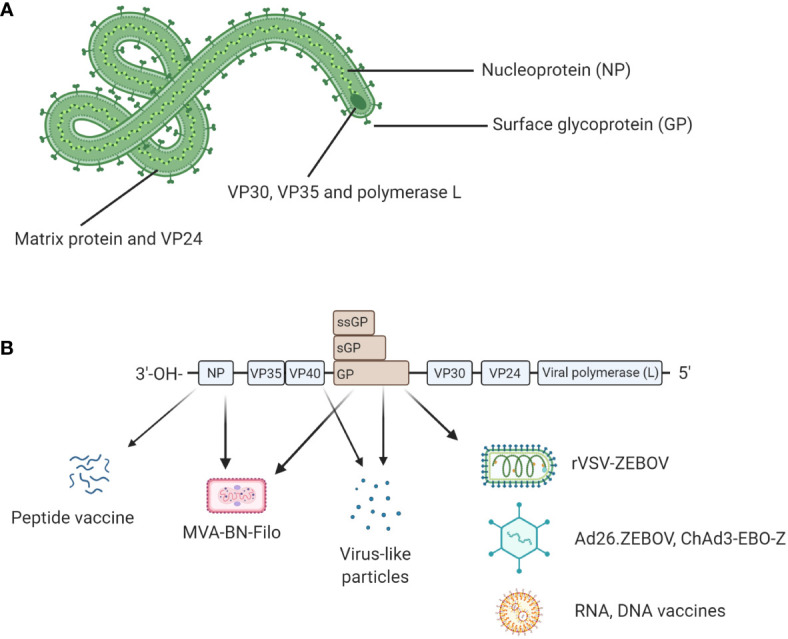
**(A)** Structural proteins of EBOV. EBOV is composed of seven structural proteins: L-protein (L), Virion protein (VP) 40, VP24, VP30, VP35, Glycoprotein (GP), and Nucleoprotein (NP). GP is exposed on the viral envelope. It mediates host cell attachment and cell entry. The nucleocapsid is composed of NP, VP35, and VP24. NP binds the viral genome. The polymerase L and its cofactor VP35 drive genome replication and transcription. VP30 is a transcription factor which is also a component of the nucleocapsid. **(B)** Genomic structure of EBOV and genetic segments targeted for vaccines. Genes encoding for each structural protein are shown. Interestingly, the GP gene also encodes for soluble GP (sGP) and small soluble GP (ssGP). Licensed vaccines (rVSV-ZEBOV, Ad26.ZEBOV) and most candidate vaccines (ChAd3-EBO-Z, RNA and DNA vaccines) use GP as antigen because antibody responses following a natural infection mainly target GP. Other antigens such as NP or VP40 have been also evaluated in the licensed MVA-BN-Filo and some candidate peptide or virus-like particle (VLP)-based vaccines. Figure created with BioRender.com.

Ebolavirus belongs to the family *Filoviridae* and consists of six characterized species; Bundibugyo (BDBV), Reston (RESTV), Sudan (SUDV), Taï Forest (TAFV), Zaire (EBOV) and the recently described Bombali (BOMV) (1) (2). The EBOV species is commonly regarded as being the most pathogenic (3) and is the focus of this review. EBOV consists of 7 proteins; L-protein (L), Virion protein (VP) 40, VP24, VP30, VP35, Glycoprotein (GP), and Nucleoprotein (NP) (4) as shown in **Figure 1A**. The virion is enveloped by membrane GP and the genome is non-segmented, with proteins encoded for by negative single-stranded RNA (-ssRNA) (4). The GP, which resides in the viral envelope, is the primary focus of much vaccine research as it is responsible for binding to the host cell and mediating cell entry. Therefore, antibodies to the GP are critical to antibody mediated neutralisation (1). NP is the major component of the nucleocapsid along with VP35 and VP24. VP40 is the matrix protein essential for budding of new virions. The RNA-dependent RNA polymerase L and the polymerase cofactor VP35 facilitate genome replication and transcription. VP30 is a component of the nucleocapsid and a transcription factor (5, 6). Typically, following attachment, EBOV will be macropinocytosed by the host cell and will escape the resulting lysosome *via* binding to Niemen Pick C1 receptor (NPC1) on the endosome membrane. This interaction results in the release of viral product into the host cell cytoplasm (7). The genome is then transcribed into seven mRNAs by the viral polymerase which consists of L protein and VP35; these products are then translated by host cell machinery. The increase in viral protein eventually results in a switch to produce and package -ssRNA. Viral proteins converge at the host cell surface where they are packaged and bud from the cell resulting in viral progeny and ultimately cell death (7).

EBOV was first classified in 1976 following an outbreak of viral haemorrhagic disease in the Democratic Republic of Congo (DRC), formally Zaire. This outbreak is thought to have originated in a missionary hospital in the village of Yambuku (8–10). Initial blood samples were sent to the Institute of Tropical Medicine (ITM; Antwerp, Belgium) where a Marburg-like virus was identified by electron microscopy (10). Samples were then sent to the Microbiological Research Establishment (Porton Down, UK) and Centres for Disease Control and Prevention (CDC; Atlanta, Georgia) where it was shown that this was a new and separate species to Marburg virus (11). This new virus was named Ebola after the local river which translates as white or clear water in the local language (8, 12). This specific species of Ebola was named Zaire after the country of origin and the archetype variant is EBOV Mayinga, named after Mayinga N`seka, a 22-year-old nurse working in Kinshasa who contracted and died from the virus. By the end of this first outbreak a total of 318 cases were recorded along with a case fatality rate of 88%. Although searched for, no animal reservoir was found from this initial outbreak and it was not until more recently that bat species were identified as a likely reservoir for the virus (13). Since 1976, there have been a number of EBOV outbreaks, one notable outbreak in 1995 occurred in Kikwit, DRC and resulted in 316 cases with a case fatality rate of 82% (14, 15). From 1996–2013, there have been a number of sporadic outbreaks of EBOV in the DRC, Republic of Congo and Gabon totalling ~700 cases and a case fatality rate of ~75% (15).

During 2014, EBOV was brought to the forefront following a large epidemic in West Africa (16, 17). During late 2013 and early 2014, a number of haemorrhagic fever cases were reported in Guékédou, eastern Guinea (16). Samples were sent to Germany & France for analysis where it was confirmed that the causative agent was EBOV (16). Unfortunately, this initial outbreak spilt over into the neighbouring countries of Liberia and Sierra Leone and by 2016, when the epidemic was declared over, had resulted in 28,646 cases and over 11,323 deaths (18). The initial spillover event is thought to have taken place in the village of Meliandou, near Guékédou where interaction with the local wildlife (e.g., bats) is thought to have resulted in transmission to humans (19). The EBOV strain responsible for this outbreak was found to have been a separate clade to those known to be circulating in the DRC or central Africa and was named Makona after the local river (20). Persistence of the virus amongst survivors and the transient but reoccurring nature of the animal reservoirs would suggest that this virus is likely still circulating within West Africa (21), therefore, future outbreaks cannot be ruled out and much research is currently ongoing to determine the prevalence of EBOV Makona amongst West African countries.

During August 2018, the world’s second largest outbreak of EBOV occurred in eastern DRC resulting in 3481 cases and 2299 deaths (22). The heightened attention these outbreaks received and the continued outbreaks in the DRC have resulted in a rapid scientific effort to both contain the virus and develop therapeutics and vaccines. The rapid research seen during the 2013–16 epidemic has similarities to the current situation that we are facing with regards to the ongoing COVID-19 pandemic, and so there are many parallels that can be drawn in terms of therapeutic and vaccine research which may help inform on the course of this and future outbreaks.

To date both the Food and Drug Administration (FDA) (23) and European Medicines Agency (EMA) have licensed the Ebola vaccine Ervebo^®^ which is based on the Vesicular Stomatitis Indiana virus (VSV) vaccine platform and manufactured by Merck. This is a viral vaccine vector that displays the EBOV Kikwit 1995 GP on the VSV capsid surface (24). Another lead candidate vaccine uses the Vaccitech Chimpanzee Adenovirus Oxford platform (ChAdOx), whereby, the EBOV Mayinga GP is incorporated into a Chimpanzee Adenovirus subgroup 3 virus; this vaccine is known as ChAd3-EBO-Z. This vaccine has been trialled as a single dose or as part of a prime-boost method with Modified Vaccinia Ankara (MVA). Due to its larger genome, the MVA boost encodes for the same EBOV Mayinga GP with additional SUDV GP, Marburg GP and TAFV NP (MVA-BN-Filo) (25). In July 2020, the EMA granted marketing authorisation for a prime-boost approach manufactured by Janssen (Johnson & Johnson) based on the recombinant Adenovirus type-26 expressing EBOV Mayinga GP (Ad26.ZEBOV) (Zabdeno^®^) and the previously mentioned MVA-BN-Filo (Mvabea^®^) in the European Union for persons greater than one year of age (26). The application of animal models in pre-clinical vaccine development and the characterisation of vaccine induced and naturally acquired immunity in humans has enhanced our understanding of potential correlates of protection for EBOV. Aspects of survivor immunology and what has been learned from animal models will now be discussed in more detail.

## EBOV Animal Models Overview

Due to the pathogenic nature of EBOV there has been a need to develop suitable animal models to study pathogenesis and vaccine efficacy. The main challenge was to develop animal models that recapitulate EVD observed in humans. The most obvious model to develop, due to cost, versatility and ethical considerations is the mouse model. However wild-type EBOV does not cause pathology in mice (27, 28). To address this problem, Bray et al. developed a susceptible mouse model to EBOV by sequentially passaging EBOV Mayinga in suckling BALB/c mice (29). The resulting mouse-adapted EBOV (MA-EBOV) now caused lethal disease in both C57B6 and BALB/c mice with death typically occurring between 4 and 6 days post-infection due to massive cytokine release followed by systemic organ damage, paralleling what is seen in humans. However, unlike human disease no haemorrhaging is seen when using MA-EBOV. Therefore, wild-type (WT) mice challenged with MA-EBOV allow for a more ethically acceptable and cost-effective way to study EVD but it does not fully recapitulate human disease (30). To address this issue, researchers have looked into using humanised mouse models which enable adoptive transfer of human peripheral blood mononuclear cells (PBMC) or CD34^+^ haematopoietic stem cells (HSC) into immune deficient mice, such as the Severe Combined ImmunoDeficient (SCID), Non-Obese Diabetic (NOD) or NOD SCID gamma (NSG) mice (31). The major benefit of these humanised models is that you are providing human target cells and probing the human immune response in an *in vivo* setting.

**Figure 2:**
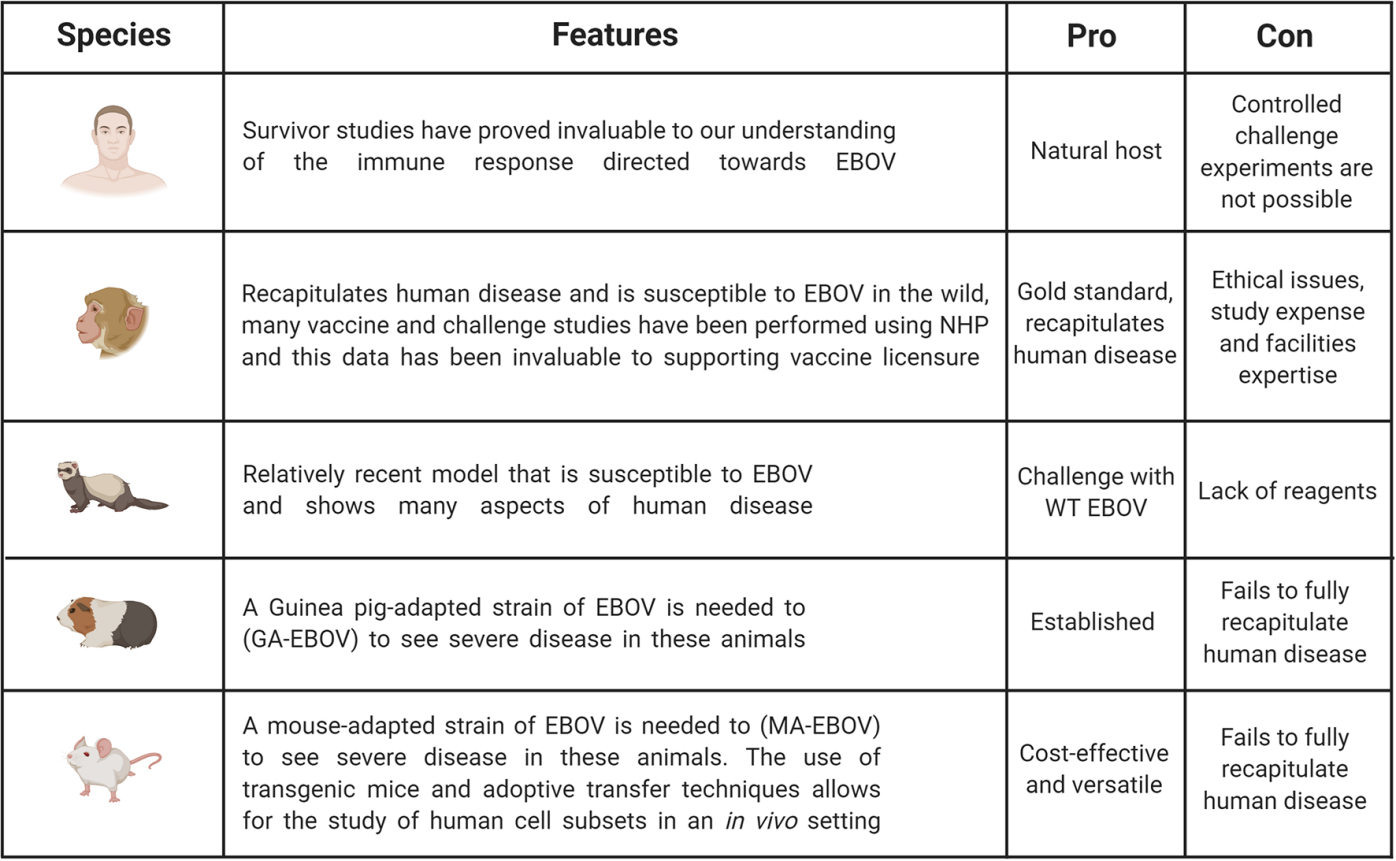
Pros and cons of animal models used in EBOV research compared to human studies. Studies in humans are invaluable to understand immunity. However, challenge experiments are only possible in animal models including non-human primates (NHPs), ferrets, Guinea pigs and mice. NHP is the gold standard as this model can be infected with WT EBOV and recapitulates EVD observed in humans. But this model is expensive and ethical and welfare concerns limit its broad use. It is the reason why other animal models have been developed even though they do not fully recapitulate human disease. Mouse models remain the most cost-effective and versatile model, but some adaptations are necessary to observe severe disease. Figure created with BioRender.

Additional models which have been used to investigate EVD are Guinea pigs and ferrets (32, 33). Guinea pigs have been used for filovirus research and although susceptible to infection, will only show mild-moderate clinical symptoms in response to wild-type EBOV (30, 32). Therefore, similar to the mouse, a Guinea pig-adapted EBOV strain was generated, in this case the disease course is closer to that seen in humans as haemorrhaging occurs (30). The ferret is a relatively new and promising animal model for EBOV. It has been demonstrated that ferrets infected intranasally with wild-type EBOV do succumb to the disease and show uncontrolled virus replications, as well as the characteristic haemorrhagic fever (34, 35). Ferrets do seem a promising model as they are susceptible to wild-type virus and show important characteristics of human disease, however an important limitation is the reduced numbers of ferret-specific reagents available. The above small animal models have been very important in pre-screening potential drugs and therapies, as well as investigating various aspects of EBOV pathogenesis, however, the gold standard animal model is the Non-Human Primate (NHP). Typically, African green monkeys, Rhesus macaques or Cynomolgus macaques have been used to study EBOV pathogenesis and the various aspects of this research are summarized below. The major benefit of NHP models is that they will more faithfully mimic the course of human disease. However, it is important to highlight the ethical concerns with regards to using NHPs, as well as the increased complexities of working at high containment and these associated costs (30). **Figure 2** illustrates pros and cons of the animal models used in EBOV research compared to human studies.

These animal models continue to have a key role in better elucidating the pathology associated with EVD, the future licensure of second generation vaccines, and in supporting general filoviruses research. We will now discuss the role these animal models in EBOV research and how these results compare with our understanding of naturally acquired immunity in EVD survivors.

## Animal Models in EBOV Research and Comparison to Naturally Acquired Immunity in EVD Survivors

### Naturally Acquired Infection

The EBOV virions major route of transmission is *via* bodily fluids and once it infects an individual it will attach and enter the host cell *via* GP binding. The GP is heavily glycosylated and has been shown to bind to a number of C-type lectins found on granulocytes, particularly DC-SIGN located on dendritic cells (DC) (36). DC and macrophages are thought to be the initial cells of infection and disruption in the normal process of both these cell types will have a serious impact on both innate and adaptive immune responses (37). In 2002, it was shown that NHPs showed a prolific release of pro-inflammatory cytokines in response to EBOV infection. It was also suggested that infection of granulocytes resulted in bystander apoptosis in a non-virus dependent manor *via* Tumor Necrosis Factor (TNF) superfamily-mediated apoptosis, further hindering the ability of the host immune system to generate an effective response (38). In addition, it has been suggested that macrophages and monocytes aid the virus by migration through the lymphatics resulting in systemic infection (39). Interestingly, human DC infected with live EBOV have been shown to have abrogated Major Histocompatibility Complex (MHC) presentation and are unable to activate T cells, whereas stimulation of immature DC with EBOV virus-like particles resulted a robust pro-inflammatory response (39). We will now discuss the adaptive immune response seen amongst survivors and various animal models.

Prior to the West African epidemic of 2013–16 there was limited information on the survivor immune response to EVD. What is known, is that studies investigating fatalities and survivors of two large EBOV outbreaks in Gabon (1996) found that survivors tended to show early and sustained levels of IgG, followed by activation of cytotoxic T cells. Whereas fatalities tended to show early T cell activation and impaired humoral responses, this was followed by a collapse in the T cell population, likely due to virus-associated apoptosis (40, 41). Wauquier et al. collected human blood samples from EVD survivors and non-survivors from EBOV outbreaks in the DRC between 1996–2003 and found that fatal outcome was associated with hypersecretion of numerous pro-inflammatory cytokines, chemokines and growth factors, however, T cell-associated cytokines appeared to have been abrogated (42). Studies on human PBMC show apoptosis of lymphocytes and death of CD8^+^ subsets and the loss of CD4^+^ subsets which was predicted to impact on the ability of the infected individuals to make a robust IgG response (43). This observation in the depletion of lymphocytes was later experimentally verified in NHP models of EVD (44). Likewise, EBOV challenge studies using Cynomolgus macaques showed, using flow cytometry, that within the PBMC compartment CD4^+^, CD8^+^, and NK cell counts decreased dramatically following the first few days of infection, in contrast CD20^+^ B cell counts remained stable. Evidence for apoptosis was seen amongst CD8^+^ and NK cell populations and it was concluded that EBOV likely blocks DC maturation thereby preventing the activation of EBOV antigen-specific T cell subsets and elimination of these subsets *via* FAS/FAS Ligand interactions (45). Similar loss of lymphocytes due to apoptosis was again later seen when using mouse models to recapitulate EVD (46). However, work has shown that adoptive transfer of moribund day 7 splenocytes into naïve mice protected these animals from MA-EBOV challenge therefore despite the commonly observed apoptosis and T cell dysregulation seen amongst fatalities, it seems that there is a functional T cell aspect which when transferred to naïve mice provides protection against challenge (47).

The comparative role of CD4^+^ or CD8^+^ T cells in protection against developing EVD has been debated. Interesting work from Gupta et al., 2004 showed that CD8^+^ deficient mice would readily succumb to non-lethal MA-EBOV infection, whereas if CD4^+^ T cells or B cell were depleted prior to challenge then mice would survive. Furthermore, using the B cell-depleted mice that survived infection, if CD8^+^ T cells were then depleted prior to re-infection then the mice would succumb to disease. This was not the case if CD4^+^ T cells were depleted prior to re-infection and suggests that memory CD8^+^ T cells alone are capable to protecting against re-infection with EBOV. Interestingly, it was also found that viral antigen could persist between 120–150 days in the tissues of mice that had their B cells depleted prior to challenge and that those mice who additionally had their CD4^+^ T cells depleted the magnitude of viral antigen that persisted was increased (48). This suggests that the humoral response is important in eliminating virus persistence, something which has been seen during the course of human disease.

Amongst human populations affected by EVD, the presence of asymptomatic individuals has been noted. Work by Leroy et al. was the first to demonstrate the potential for EBOV asymptomatic infection. Indeed, they identified individuals who mounted a strong and effective pro-inflammatory response to the virus. Furthermore, they demonstrated that following this early response there is cytotoxic T cell activation followed by an EBOV-specific IgG response (49, 50). Serological surveys have also played an important role in the understanding of EVD amongst human populations. One such survey using an IgG ELISA to measure the GP-specific response amongst rural villages in Gabon measured >4000 individuals over 3 years and found that 15.3% of participants showed prior exposure to EBOV; this was higher amongst the forested regions where ~20% of people showed exposure to EBOV (51). This finding that the seroprevalence amongst the rural populations is greater that the urban area has again been shown in the DRC (52).

Following a large outbreak of EBOV in 1995, located primarily in Kikwit, DRC, a number of studies investigated the humoral response to EVD amongst survivor cohorts. Maruyama et al., 1999 generated a panel of human monoclonal antibodies using survivor PBMC samples from the Kikwit outbreak and phage display technology (53, 54). This resulted in the generation of the widely used human Monoclonal antibody (MAb) KZ52 which was subsequently shown to protect Guinea pigs from challenge with EBOV (55).

The West African epidemic of 2013–16 fuelled a significant EBOV research programme. It led to the largest cohorts of survivors, which strengthened international efforts to understand host immune responses following naturally acquired infection. Several longitudinal studies were initiated at that time. Adaptive immune responses were dissected in survivors in the field and in western repatriated patients. In addition, animal models enabled the assessment of the protective role of specific immune components.

The antibody response following EBOV infections in humans has been described in several studies. MAbs isolated from EVD survivors of previous outbreaks in Africa as previously mentioned (53–57) or the 2013–2016 West African epidemic (58–60) were shown to be neutralizing and protective in animal EBOV challenge models. Bornholdt and colleagues isolated 349 GP-specific MAbs from the peripheral B cells of a convalescent donor who survived the 2014 EBOV epidemic. They found that 77% of MAbs were able to neutralize live EBOV. They also showed that MAbs which targeted the GP stalk region proximal to the viral membrane, inhibiting cleaved virus in endosomes, were particularly efficient to protect mice against lethal EBOV challenge (59). Interestingly, some MAbs isolated from this library showed pan-neutralizing and protective capacities in mice and ferrets (58). Additional MAbs isolated from 2013–2016 West African epidemic and 2018 outbreak in the DRC targeting epitopes at the base region of the GP also demonstrated pan-neutralizing and protective capacities in mice, Guinea pigs and ferrets (60). Similar findings were observed in some survivors from the 2014 Boende EVD outbreak who mounted pan-ebolavirus responses but also pan-filovirus neutralizing responses (61). Recently, Dowall et al. investigated whether the established human serological reference standard (the 1st WHO International Standard) for Ebola virus antibody, could be used to provide a quantifiable correlate of immune protection *in vivo*. Dilutions of the serological standard were administered to groups of Guinea pigs through intraperitoneal route in comparison with one control group. One day later, all animals were challenged with a lethal dose of EBOV *via* subcutaneous route. They observed that only animals receiving the highest dose of the serological Standard exhibited evidence of delayed progression of disease (62). This standard may be very valuable for evaluation and prediction of protective humoral responses in vaccination studies.

Some studies have analyzed antibody isotypes generated post-EBOV infection and dissected the role of B cells following acute infection. Gunn et al. showed that GP- and secreted GP (sGP)-specific antibody responses were mounted in 14 survivors from Sierra Leone. Interestingly, most survivors developed neutralizing EBOV-specific IgG1 and IgA with innate immune effector functions (63). The role of antibodies and B cell responses in the initial control of infection was also studied in western repatriated patients even though intensive and/or experimental care used in high-income countries might influence their immune responses. Four acute *Ebolavirus*-infected patients during the 2014 West Africa epidemic and repatriated to Emory University hospital (USA) were enrolled for a longitudinal study analysing B cell responses to Ebola virus infection. Davis et al. found that IgG1 persisted over time, IgG3 declined early and IgG4 appeared late. Following the recruitment and activation of B cells, they observed that EBOV infection induced changes in the antibody repertoire. Only a small subset of antibodies was able to recognise cell-surface GP but this subset contained all identified neutralizing antibodies. Interestingly, they also reported conserved neutralizing antibody rearrangement across donors (64). Williamson et al. demonstrated that human B cell responses between 1–3 months post-recovery were characterized by a low frequency of EBOV-specific B cells encoding for antibodies displaying low neutralizing activity even though one neutralizing antibody human antibodies isolated in this study led to protection in a mouse EBOV challenge model (65). Khurana et al. analyzed longitudinal human antibody repertoire against viral proteome from an Ebola virus survivor from Sierra Leone evacuated to USA and treated on a randomised controlled clinical trial comparing an immunotherapy plus standard of care to standard of care alone. This patient had randomised to receive standard of care treatment alone. They found long-lasting IgG/IgM/IgA epitope diversity. During the acute phase, antibodies predominate to VP40 and GP. One year post-onset of symptoms and despite undetectable virus, a diverse IgM repertoire against VP40 and GP epitopes was observed suggesting occult viral persistence. Finally, they described specific sites in C terminus of GP1 and GP2 which were immunogenic following immunisation in rabbits leading to neutralizing antibody induction which were protective in a lethal EBOV challenge mouse model (66).

Following the West African epidemic of 2013–16, cellular responses have also been analyzed in more detail amongst survivor cohorts and among repatriated western survivors. Ruibal and colleagues evaluated T cell immune responses in EVD patients at the time of admission to the Ebola Treatment Centre in Guinea and longitudinally until discharge or death. They found that patients with elevated levels of T cell inhibitory molecules PD-1 and CTLA-4 expressed on CD4^+^ and CD8^+^ T cells were more likely to succumb to disease. These parameters correlated with elevated inflammatory markers and high virus load (67). In another study, the same team demonstrated that T cell responses in fatal patients were oligoclonal and did not result in viral clearance. Contrary to fatal cases, survivors developed highly diverse T cell responses and maintained low levels of T cell inhibitors, which led to viral clearance (68). Other studies analyzed EBOV antigens leading to cellular responses. In 2018, work by Sakabe et al. was focused on the Ebola-specific CD8^+^ T cell responses in individuals infected during the 2013–2016 epidemic in Sierra Leone. They examined T cell memory responses to GP, sGP, NP, VP24, VP30, VP35, and VP40. They found that CD8^+^ responses to the NP were immunodominant (69). Herrera et al. developed an anthrax toxin-based Enzyme-Linked ImmunoSpot (ELISPOT) assay to analyze T cells responses in 19 EVD survivors, 10 asymptomatic individuals who were known to be closed contacts with symptomatic EVD patients and six control healthcare workers in Nigeria during 2017. They found that seropositive asymptomatic individuals mounted stronger IFNγ and TNFα responses to NP, VP40, and GP1 EBOV fusion proteins compared to the EVD survivors (70). Consistent with Sakabe and colleagues’ study (69), cellular responses directed to the EBOV NP were strongest in comparison with other EBOV antigens in survivors and asymptomatic individuals (70). Interestingly, Lavergne et al. made a connection between post-Ebola syndromes observed in patients in the field and T cell responses. The impact of the persistence of T cell responses on post-Ebola syndromes in 37 survivors in Sierra Leone was analyzed and they demonstrated that survivors with arthralgia and ocular symptoms had a significantly higher EBOV-specific CD8^+^ and CD4^+^ T cell responses compared to survivors without any sequelae (71). Recently, we reported in a longitudinal study that T cell and neutralizing antibody responses were long lived in EVD survivors in Guinea. We determined that the dominant CD8^+^ polyfunctional T cell phenotype was IFNγ^+^, TNF^+^, IL-2^-^ (72). We further characterized T cell epitopes to the EBOV GP and we found that survivors generally responded to a portion of the receptor-binding domain. We found that both CD4^+^ and CD8^+^ T cells contributed to specific T cell memory but with a different cytokine profile. CD4^+^ T cells produced IFNγ, TNFα, and IL-2 whereas CD8^+^ T cells only produced IFNγ and TNFα (73). Longitudinal analyses in western repatriated patients infected during the 2013–2016 West Africa epidemic also gave new insights about cellular immune responses. Agrati and colleagues studied immune responses in two western patients repatriated to Italy. They showed a reduction in CD4^+^ T cell frequency and an increase of CD8^+^ T cell frequency during the vireamic period (74). This inversion of CD4/CD8 ratio was reverted during the recovery period of these patients and interestingly, this inversion had not previously been observed in mouse models (46). They also found a significant T cell activation and an enhanced PD-1 expression, as well as an impaired IFNγ production which was associated with virus reactivation (74). McElroy et al. investigated the cellular response to four previously-mentioned acute *Ebolavirus* infected patients at Emory University hospital, where they noted a robust T and B cell activation in these four patients. A high percentage of activated CD4^+^ and CD8^+^ was also observed up to 60 days after symptom onset. They found activation of both CD4^+^ and CD8^+^ T cells to several Ebolavirus proteins. They observed consistent IFNγ responses following stimulation with NP peptide pools. The strongest responses were CD8^+^ T cell-mediated and directed against the Ebola virus NP (75); this correlates with Sakabe and colleagues’ findings in survivors in Sierra Leone (69). Dahlke et al. described more specifically the persistence of T cell activation beyond viral clearance in a western repatriated patient at the University Medical Centre Hamburg-Eppendorf. At days 37 and 46 after illness onset, GP-specific T cells were especially found in the CD8^+^ T cell population and were detectable at low magnitude. The largest fraction of EBOV GP-specific T cells revealed an overall low IFNγ, IL-2, and MIP-1β responses upon stimulation with GP peptide pools. However, they observed that the largest fraction of this cell population produced TNFα and expressed the degranulation marker CD107a (76). These findings contrast with previous findings found in NHPs showing a peak of IFNγ and IL-2 expression by CD8^+^ T cells at day 14 post-infection (77).

Finally, key transcriptomic studies informed on correlates of protection and gave new insights about predictors to patient outcomes. Transcriptomic immune signature was analyzed by Liu et al. in blood samples from 112 infected Guinean patients in 2014 and 2015 who survived or succumbed to EVD. These samples were taken during EBOV diagnosis where blood was analyzed by RT-PCR in the Ebola Treatment Centre. Subsequent transcriptomic analysis showed that fatal patients displayed significant elevated levels of IFN and acute phase response signalling compared to survivors during the acute phase of infection. An upregulation of albumin and fibrinogen genes was also observed in fatal cases suggesting significant liver pathology. Interestingly, this study demonstrated that there was an increase of NK cell populations in survivors (78). A similar gene expression profile study was performed with 44 survivors or fatal EVD patients in Sierra Leone between 2014 and 2016. They observed a dysregulation in inflammatory responses in fatal cases compared to survivors in the late phase of disease. In addition, a strong positive correlation between the upregulation of inflammatory mediated and EBOV viremia was observed. They also found that survivors developed anti-IgM and anti-IgG responses earlier and to a greater extent than fatal patients (79). Similar transcriptional analysis can be performed in animal models like NHPs and ferrets whose sequence genome was published in 2014 (80). Cross et al. compared transcriptomics in Ebola Makona-infected ferrets, NHPs and humans showing strong similarities between pro-inflammatory and pro-thrombotic programs induced in the species analyzed in this study (81). RNAseq technologies were rapidly developed and diversified in the last decade. It is now an essential tool to pursue dissection of immune responses in the context of Ebola virus infections in humans and animal models.

Collectively, the data generated in survivors and animal models for more than two decades have significantly helped in identification of host immune responses following Ebola virus infection. However, a better link should be made between these numerous findings to understand the complex interplay between innate and adaptive immunity. NK cell subsets, T and B cell memory responses could be dissected in more detail. RNAseq technologies will also be crucial to analyze T cell receptor and B cell receptor repertoires. Humanised mice recently developed are also an efficient tool in the pursuit of a search for correlates of protection. Finally, most studies of adaptive immunity have been largely performed in the context of survival during the immediate aftermath of acute infection. However, it was observed that hundreds of long-term survivors experience a range of chronic symptoms which are still poorly understood (82).

The sum of these results also significantly helped in therapeutics and vaccine development. The 2013–16 West African epidemic highlighted the urgent need to produce and assess a safe and effective Ebola vaccine for humans (83).

Immune responses to EBOV infection in animal models and human survivors detailed in this section are summarized in the **Table 1**.

**Table 1:** Immune responses to EBOV infection in animal models and human survivors.

Model	Responses to EBOV infection				
	Clinical Presentation	Antibody production and protection	CD4^+^/CD8^+^	B cells	NK cells
**Human survivors**	Range from asymptomatic to flu-like symptoms with continued progression to: vomiting, diarrhoea, rash, kidney and liver damage, haemorrhaging and low white blood cell (84).	Strong and early humoral response is associated with survival (78) (72).	Strong and sustained T cell responses, with reduced expression of inhibitory molecules and a diverse T cell repertoire, are associated with survival (67) (68). Persistent T cell responses are associated with sequalae (71) (72) (73).	Extensive activation during acute infection. Subclass composition changes over time with persistent IgG1, rapid decline of IgG3 and late increase in IgG4 (64).	Increase in survivors and decrease in fatalities (78).
**Macaques (cynomolgus and rhesus)**	Identical to human EVD (85).	Antibodies produced in response to infection contribute to survival (86).	Both CD4^+^ and CD8^+^ counts decrease during acute infection stage (45).	Levels in blood remain stable (45).	Decline during acute phase (45) (87).
**African green monkeys**	Identical to human EVD. Rash is less frequent (85).	Protection from natural infection is difficult to determine due to lack of NHP model for mild EVD (88).	Both CD4^+^ and CD8^+^ cells show signs of apoptosis and depleted levels in lymph nodes. CD4^+^ cells increased in germinal centres (44).	Lymphocyte depletion in B cell follicles (44).	ND
**Mice (mouse-adapted model)**	Mouse-adapted virus and intraperitoneal injection required for infection. Unlike humans, EVD does not manifest with rash, coagulopathy or haemorrhagic symptoms. However they can succumb to disease (85).	Protection from antibody transfer has been reported (59) (60) (65).	T cells show drastic depletion during the acute phase (46) but are still important for protection. CD8^+^ T cells provide protection against acute infection (47) whilst CD4^+^ T cells confer long-term protection and challenge viral persistence (48).	Important for fighting viral persistence (48).	Drastic depletion in blood during acute phase (46) but accumulation in EBOV infected tissues (89). Differential effects depending on viral load (89).
**Naïve or immunocompromised mice**	Unlike the mouse-adapted model, EVD in this model can manifest with haemorrhagic symptoms (85).	Acquire protection from antibody transfer (90).	Adoptive T cell transfer from vaccinated or infected mice can confer protection against MA-EBOV (91) (47).	ND	NK cells are required for protection against infection, even if they have received prior protection from VLPs (92).
**Humanised mouse models**	Various models capable of recapitulating human EVD (85). Can mimic human EVD, including symptoms of hepatic pathology, lymphocyte apoptosis, haemorrhaging, and lethal outcome when infected with MA-EBOV (88) (93) (94).	ND	T cell environment varies depending on model. Restricted CD8^+^ responses reported, with limited MHC interactions (95). CD8^+^ (and presumed CD4^+^) T cell activation observed 8 days after inoculation with EBOV (96).	Can show measurable B cell responses (88) (88) but the B cell compartment is short-lived (95).	Important in early immune response and shows a significant decrease 5 and 8 days after inoculation (96).
**Ferret**	Can be infected with wild-type EBOV and demonstrate human EVD symptoms including rash, coagulation abnormalities and haemorrhaging, not seen with some other models (34) (35).	Acquire protection from antibody transfer (58) (60).	ND	ND	ND
**Guinea pig**	Require Guinea pig-adapted virus. Does not recapitulate rash or haemorrhagic symptoms but EVD does manifest with platelet reduction, liver pathology, and lymphocyte apoptosis (85).	Acquire protection from antibody transfer (55).	ND	ND	ND
**Hamster Model**	Requires adapted EBOV. Recapitulates key features of EVD including coagulopathy and haemorrhaging, absent in many rodent models (97) (98) (85).	Acquire protection from antibody transfer (99).	CD4-dependent antibody responses (99).	ND	ND

Clinical presentation and adaptive immune responses observed in each species. NA, Not applicable; ND, Not determined.

### Vaccination

On August 8, 2014, the WHO declared the epidemic in West Africa a global health emergency which coincided with rapid development of an effective Ebola vaccine which built on efforts initiated in the 1990s. Vaccines which were successfully licensed are based on viral vectors. However, vaccine candidates based on other strategies have been tested in preclinical trials or even in early stage of clinical trials. As shown in **Figure 1B**, most Ebola vaccine strategies are based on GP as antigen given it has been shown that neutralizing antibodies produced after a natural infection mainly targeted EBOV GP in humans and animal models. However, antibody responses and cellular responses specific to NP and VPs have been also described in some studies. Thus, NP or VP40 have been also evaluated in some vaccine formulations.

The next section will discuss vaccination studies in animal models and humans which provided key information on the role of the cellular and humoral immune responses with regards to EBOV pathogenesis and protection.

#### Animal Studies

Early animal studies performed by Olinger et al. showed the importance of the cellular immune response by vaccinating WT C57B6 or BALB/c mice with Venezuelan equine encephalitis virus replicons (VRP) which had been genetically modified to express GP, NP, VP24, VP30, VP35, or VP40 proteins. They found that murine antigen-specific T cells to NP and GP were readily generated and if these antigen-specific T cells were expanded *in vitro* they could protect naïve mice from EBOV challenge when adoptively transferred (91). A potentially important role for CD4^+^ T cells was demonstrated in 2002 when mice and NHPs were vaccinated against EVD using liposome-encapsulated irradiated EBOV. It was found that if during or prior to vaccination mice had their CD4^+^ T cell compartment depleted using antibodies, then the protective effect of vaccination was abrogated and mice succumbed to infection with MA-EBOV (100). The finding, that CD4^+^ T cells play a key role in protection against EVD following vaccination, was more recently shown by Marzi et al. In a similar fashion, they depleted the CD4^+^ population from NHPs prior to vaccination with rVSV and concluded that the CD4^+^ population was critical for establishing an IgG specific response (101). Seminal evidence for the importance of T cells to EVD survival comes from the work of Sullivan et al. who vaccinated NHPs with human recombinant adenovirus serotype 5 (rAdHu5) which encoded for EBOV GP. Cynomolgus macaques were vaccinated then exposed to EBOV. Interestingly, if post-vaccinated animals underwent T cell depletion using an anti-CD3 MAb, they lost their ability to control disease and succumbed to infection. Furthermore, if prior to challenge primates were CD8^+^ T cell depleted using a MAb then, again, they were unable to control disease, this was not the case for CD4^+^ T cell depletion prior to challenge (102). Additionally, using NHPs, it has been suggested that protection resulting from Adenoviral vectors requires the generation of effector memory CD8^+^ T cells that produce both IFNγ and TNFα and that durable protection, as shown in NHPs, require CD8^+^ memory T cells that are polyfunctional for IFNγ, TNFα, and IL-2 (103).

Using Guinea pigs and mice to investigate various vaccination routes, it was shown that vaccination *via* the sublingual route with an adenoviral (Ad5) vector provided a cellular immune response that was unaffected by pre-existing immunity to the viral vector. Therefore, making it more likely that this vector and delivery route could be used multiple times for the same host. Additionally, both mice and Guinea pigs were protected from lethal challenge (104).

Pre-existing immunity to the viral vector is a concern for successful vaccination and the implications of this have been modelled using animal studies. For example, Choi et al. found that adoptive transfer of splenocytes from animals previously vaccinated with the empty viral vector (Ad5) followed by immunisation with the Ad5-EBOV vaccine showed abrogation in CD8^+^ T cells responses and reduced GP specific IgG1 responses. However, if vaccination was given *via* a different route to that of the empty vector, CD8^+^ T cell responses were not abrogated. This importantly highlights the role of pre-existing immunity to eliciting the optimum immune response to EBOV (105). Early use of adenovirus vectors for vaccination against EVD came from Roy et al, who used simian Adenovirus type-22 and -21 and compared the responses with the human Ad5 platform in an attempt to circumvent any issues with vaccinating human populations already exposed to human Ad5. It was found that both mice and NHPs mounted B and T cell responses and that mice were protected from MA-EBOV challenge following vaccination (106).

Initial preclinical work using VSV as a vaccine platform demonstrated that antibodies were sufficient to protect mice from infection after immunisation with VSV expressing Zaire EBOV Kikwit 1995 GP (rVSV-ZEBOV), while the depletion of CD8^+^ T cells did not compromise protection (107). Feldmann et al. tested the efficacy of rVSV-ZEBOV vaccine in post-exposure treatment in Rhesus macaques and found that 4/8 macaques were protected if treated up to 30 min following a lethal infection. They compared immune responses between the macaques which survived and those ones which succumbed. They observed that neutralizing antibodies were detected on days 14–36 after challenge in animals that survived the challenge, while humoral response was not detected in animals that succumbed to the challenge, suggesting a major role of the humoral response. They also observed a decline of CD4^+^ and CD8^+^ in most macaques regardless of treatment and outcome. The only difference noticed in cellular responses was the percentage of NK cells which did not decrease but actually increased in most macaques treated with the vaccine (86). Geisbert et al. analyzed rVSV-ZEBOV immunogenicity and protective efficacy in a macaque model challenged with ZEBOV by aerosol. All vaccinated primates were protected from challenge, and they found that immunisation induced ZEBOV-specific IgG responses which increased following the challenge but there was no evidence of IFNγ or TNFα production in CD4^+^ or CD8^+^ before or after the challenge (108). The same team also showed that in immunocompromised NHPs the vaccine was protective and well tolerated. Here, Rhesus macaques previously infected with simian-human immunodeficiency virus were vaccinated with rVSV-ZEBOV. The vaccine produced no serious side effects. However, when challenged with EBOV, 2/6 of these vaccinated animals succumbed to disease; interestingly those animals that died had lower CD4^+^ counts (109). Investigations into the mucosal response to vaccination against EVD found that NHPs vaccinated intranasally with rVSV-ZEBOV were protected against challenge, and this route seemed to be more potent than intramuscular injection. These findings will have important implications to vaccination amongst human populations (110). Finally, Konduru et al. made an Fc fusion protein consisting of the extracellular domain of EBOV GP and human IgG1 (ZEBOVGP-Fc). Mice vaccinated with ZEBOVGP-Fc showed both cellular and humoral responses and were protected against challenge with MA-EBOV (111). Demonstrating that vaccine platforms do not necessarily need to be viral vector based. Some studies have attempted to compare the immune correlates elicited in both murine and primate experiments. Here, Wong et al. vaccinated immunocompromised mice and found that vaccine induced protection was primarily B and CD4^+^ T cell mediated. They also demonstrated that Guinea pig and NHP showed a strong correlation between survival and GP-specific IgG. This suggests that the magnitude of the GP-specific IgG response is a meaningful correlate of protection (112). A study also analyzed the long-term protective efficacy of rVSV-ZEBOV in murine and Guinea pig models. While they observed 100% survival of Guinea pigs challenged with a lethal dose of adapted EBOV 12 and 18 months post-vaccination, only 80% of mice survived at 12 months post-vaccination. Interestingly, they measured higher EBOV GP-specific IgG responses in mice which survived up to 12 months post-vaccination, suggesting a major role of antibody responses in protection (113).

#### Clinical Trials

Preclinical studies were the foundation of the world’s first Zaire Ebola vaccine rVSV-ZEBOV (brand name Ervebo^®^) approved by the EMA and FDA in 2019. Following animal studies, between 2015–2017, the rVSV-ZEBOV vaccine was also studied in Phase 1 clinical trials (114–117) and a Phase 3 safety and manufacturing-consistency clinical trial (118) in North America, Europe, and Africa. Even though the rVSV-ZEBOV has the potential for some adverse effects (119, 120), the sum of these data showed an acceptable immunogenicity and safety profile. Antibody response was measured in 89%–100% of vaccinated individuals for at least 24 months (121). One study was able to provide data on clinical efficacy. This Phase III study was conducted in Guinea using the approach of ring vaccination and in place of a placebo control, the rings were randomly assigned to either ‘immediate’ vaccination (in a defined timeframe of 24 h), or vaccination after a 21-day delay. In this study, Henao-Restrepo et al. reported 100% efficacy of the vaccine. Among 2,119 people who received the vaccine immediately, no cases were reported in a period of 11 days, whereas 16 cases were identified within the same time frame among 2,041 people in the delayed vaccination group (122). However, the protocol revealed there may be a bias with respect to the intervention of medical team in the immediate or delayed vaccination groups. The presence or absence of the medical team was not identical in the vaccinated groups and may have potentially influenced outcomes (123). Furthermore, indirect evidence from a randomised controlled trial of EVD therapeutics in DRC showed that a portion of clinical EVD cases had received vaccination. 25.0% reported that they had received the vaccine; of these, 38.7% reported that they had received the vaccine at least 10 days before the onset of clinical symptoms (124). Safety and immunogenicity of rVSV-ZEBOV were also studied following high-risk exposure. In 2015, rVSV-ZEBOV was used on a laboratory worker, 43 h after a high-titre needlestick injury. The physician developed fever and moderate-to-severe symptoms 12 h post-vaccination, but symptoms disappeared over 3 to 4 days. Activation of T cells and plasmablasts were detected early post-vaccination. The peaks of EBOV GP-specific IgG and IgM, as well as an increase of cytokine-producing CD4^+^ and CD8^+^ T cells were observed on day 17. Antibodies against VP40 (not in the vaccine) were not detected, suggesting that the immune responses are not due to natural EBOV infection (125). For 1 year, Davis et al. followed 45 people who came into direct contact with a healthcare worker presenting a late reactivation of EVD and who were elected to receive rVSV-ZEBOV. Three months following vaccination, 100% of individuals had seroconverted. In addition, neutralizing antibodies were detected in 73% of volunteers 12 months post-vaccination. Nobody exposed to the virus became infected. No severe vaccine-related adverse events were reported. However, common side effects associated with vaccination were characterized by fatigue, myalgia, headache, and arthralgia. Interestingly, arthralgia, myalgia and fatigue occurring at the time of study were associated with a higher proportion of CD8^+^ IFNγ and CD4^+^ IL-2-secreting cells, while headache was associated with higher CD4^+^ IL-2 and IFNγ ELISpot responses (126). The rVSV-ZEBOV vaccine has been the most studied in animal models and clinical trials as prophylaxis or following high-risk exposure. Even though this one-dose vaccine was shown to be successful in the field, its level of efficacy in humans remains to be confirmed and correlates of protection to be clarified. **Table 2** summarizes protection and adaptive immune responses observed in humans and animal models following rVSV-ZEBOV vaccination.

**Table 2:** Responses to rVSV-ZEBOV.

Model	Responses to rVSV-ZEBOV				
	Protection?	Antibody production?	CD4^+^/CD8^+^	B cells	NK cells
**Humans**	Yes (127).	Yes. Antibodies also cross-react with other EBOV species (127).	Increased activation of both CD4^+^ and CD8^+^ T cells (76).	Polyclonal, convergent B cell responses observed in a trial of 4 vaccinees (127).	Modulation of NK cells contributes to early efficacy of the vaccine (128).
**Macaques (cynomolgus and rhesus)**	Yes (86) (108) (113).	Yes. Strong correlation observed between GP-specific IgG and survival (86) (108) (113).	Similar to natural infection, circulating CD4^+^ and CD8^+^ T cells declined during infection, regardless of prior treatment or vaccination (86). CD4^+^ T cells likely contribute to establishing protection from vaccination (109) whilst CD8^+^ T cells are not required for vaccine protection (101).	Required for production of antibodies which mediate protection. Although CD20^-^depleted macaques were shown to have detectable EBOV-specific antibodies, this is likely due to persistence in lymphoid organs (101).	Increased in response to vaccine (86).
**African green monkeys**	ND	ND	ND	ND	ND
**Mice (mouse-adapted model)**	Yes (129) (113).	Yes. Strong correlation observed between GP-specific IgG and survival (113).	CD8^+^ T cell depletion does not compromise protection (107). CD4^+^ T cells (and B cells) are main mediators of protection (112).	B cells (and CD4^+^ T cells) are the main mediators of protection (112).	NK-cell population is enhanced by vaccine and increases survival (130).
**Naïve or immunocompromised mice**	Yes (107).	Able to tolerate vaccine (107).	NA	NA	NA
**HSC-transfer mice**	ND	ND	ND	ND	ND
**Ferret**	Yes (131).	Yes (131).	ND	ND	ND
**Guinea pig (guinea-pig adapted model)**	Yes (112).	Yes. Strong correlation observed between GP-specific IgG and survival (112).	ND	ND	ND
**Hamster**	Yes (24).	Yes (24).	ND	ND	ND

Protection and adaptive immune responses observed in humans and animal models. NA, Not applicable; ND, Not determined.

In July 2020, the European Commission granted Marketing Authorisation for the two-dose vaccine regimen Ad26.ZEBOV (Zabdeno^®^) and MVA-BN-Filo (Mvabea^®^). This is a multivalent prime-boost vaccine based on an Adenovirus type 26-vectored vaccine encoding Ebola virus GP boosted by a MVA-vectored vaccine encoding GP from Ebola, Sudan and Marburg viruses, as well as the NP of Tai Forest virus. In a Phase I study of healthy volunteers in the UK, vaccination with Ad26.ZEBOV and MVA-BN-Filo did not result in any vaccine-related serious adverse events. All vaccine recipients had EBOV GP-specific IgG detectable 21 days post-boost and at 8-month follow-up. Within randomised groups, at 7 days post-boost, at least 86% of vaccine recipients showed Ebola-specific T cell responses (132). Another Phase I trial performed in Oxford showed that this two-dose vaccine could confer immunity for at least 360 days and was well tolerated (133). In 2019, Anywaine et al. and Mutua et al. published the results of two Phase I trials performed in Uganda and Tanzania, as well as in Kenya, respectively. Both trials showed good immunogenicity and safety profiles in healthy volunteers (134, 135). Phase II and III clinical trials in various cohorts of participants (e.g., healthy, elderly, HIV, children) have been recently completed or are currently in progress in Africa and Western countries (**NCT02564523, NCT04228783, NCT03929757, NCT02598388, NCT04028349, NCT02509494, NCT02661464, NCT02543268**). To assess immunogenicity of the vaccine, most clinical trials analyzed EBOV GP-specific IgG and sometimes neutralizing antibody levels. Based on current information available on ClinicalTrials.gov, only one clinical trial is evaluating plasma cytokines/chemokines and T cell responses (**NCT04028349**). Even though the efficacy remains to be determined in humans, this Ebola vaccine has been deployed in the North Kivu region of the DRC, following recommendation from the WHO’s Strategic Advisory Group of Experts (SAGE), and in Rwanda, following conditional approval in 2019 under an “exceptional emergency”, as part of outbreak containment efforts in the region (136). However, a two-dose regimen is less suitable for an outbreak response where immediate immune protection is essential. This two-dose vaccine maybe a more suitable strategy for healthcare professionals, frontline workers or visitors who plan to go to areas with an ongoing EVD outbreak.

Another lead candidate is a vaccine named ChAd3-EBO-Z. This vaccine is based on chimpanzee Adenovirus subgroup 3 (ChAd3) vaccine encoding EBOV Mayinga GP. Stanley et al. demonstrated that a chimpanzee-derived replication-defective adenovirus vaccine was able to generate protection against acute lethal EBOV challenge in macaques and after a boost with MVA, they observed a durable protection against lethal EBOV challenge (137). Following this success in the NHP model, the vaccine evaluation advanced into clinical trials. Phase I and II trials investigated this vaccine on its own (138, 139) or in combination with an MVA boost (25, 140) or MVA-BN-Filo (141). The main specificity of this chimpanzee adenovirus platform is the induction of exceptional antigen-specific T cell responses previously observed in animal models (142) and humans (143) in the context of the development of vaccine against malaria. Similar results were shown in the context of Ebola vaccine. The ChAd3-EBO-Z vaccine boosted with MVA was shown to be safe (141) and to elicit EBOV-specific antibody and T cell immune responses superior to those induced by the ChAd3 vaccine alone (25). The single-dose ChAd3-EBO-Z was tested side-by-side with the single-dose rVSV-ZEBOV in Phase II trial in Liberia. In this study, antibody responses were slightly lower with ChAd3-EBO-Z vaccine compared to rVSV-ZEBOV. However, post-vaccination symptoms like headache, muscle pain or feverishness were less frequent with ChAd3-EBO-Z than post-vaccination with rVSV-ZEBOV. Unfortunately, T cell responses were not evaluated in this study (144). The efficacy of ChAd3-EBO-Z alone or in combination with MVA boost remains to be confirmed in humans.

Human and animal research demonstrate the importance of both B and T cell immunity in protection from EBOV infection. The current lead vaccines demonstrated that they were able to induce EBOV-specific antibody responses. However, the duration of antibody responses, as well as the generation of memory B cell responses remain to be confirmed. Lead candidate vaccines previously described have also been shown to induce T cell responses. It is likely that the chimpanzee adenovirus platform is more suitable to induce strong antigen-specific T cell responses. However, as described in this review, the level of T cell responses might play a role in vaccine-associated side effects. Another crucial point is the presence of EBOV-NP specific T cell responses, especially CD8^+^ T cell responses detected in survivors and even asymptomatic individuals, which could suggest an important role of NP antigen in protection. Currently, the lead Ebola vaccines are focused on EBOV GP as the antigen. Thus, NP should be included in vaccine formulation along with the EBOV GP as it might boost cell-mediated immunity. Finally, even though rVSV-ZEBOV, Ad26.ZEBOV- MVA-BN-Filo, ChAd3-EBO-Z generated strong immunogenicity and showed success in the field, to what extent B and T cell immunity is required for protection is still unclear. Consequently, immunogenicity and protection provided by vaccines must be compared to immunity in survivors. Some recent studies compared side-by-side immune responses following a natural infection versus vaccination. Fuentes et al. compared antibody responses in plasma from three Western survivors from the 2014 West Africa epidemic (2-6 months post-infection) who received experimental treatments, as well as a pool of 6 plasma from Sierra Leonean convalescents who did not receive experimental treatment versus pools of plasma from ChAd3-MVA vaccinees in the UK (2-12 months post-vaccination). One pool had low and the other one had high neutralization titres. They found higher antibody responses and stronger antibody affinity in survivors, as well as high neutralization titres compared to vaccinees. Survivors demonstrated IgG-dominant antibody responses whereas a predominant IgM response was detected after vaccination. Natural EBOV infection generated a more diverse antibody epitope repertoire compared to vaccination. They also observed that antibodies preferentially recognised antigenic sites in specific GP2 domains (the fusion peptide and HR2) in survivors than in vaccinees and that this was associated with neutralization titres (145). Another study compared GP epitopes bound by anti-EBOV GP antibodies following a natural EBOV infection versus vaccination with rVSV-ZEBOV. They analyzed the sera from seven vaccinees with rVSV-ZEBOV and one western EVD survivor who contracted an Ebola virus infection in Sierra Leone in 2014 and was repatriated to Germany. An epitope mapping approach showed that IgG and IgM antibodies from the survivor or the vaccinees bound different epitopes in the EBOV GP (146). Koch et al. compared the functionality of anti-EBOV GP antibodies between 10 rVSV-ZEBOV vaccinees from the Phase I clinical trial in Hamburg 6 months post-vaccination and 25 EVD survivors 12 months after discharge. They did not find any significant differences between the levels of circulating Ig subclasses. However, they observed a higher neutralization capacity of plasma samples from survivors than that of vaccinee samples, as well as a higher capacity to induce cellular responses. They also determined that the levels of IgG1 positively correlated with virus neutralization in survivors but not in vaccinees (147). The sum of these studies suggest that vaccines may induce different immune responses in vaccinees from those observed in survivors. However, it is essential to keep in mind that the quality of immunity might be also affected by the vaccine vector. Meyer et al. tested various human and avian paramyxoviruses expressing EBOV GP and demonstrated different serum antibody profiles in a Guinea pig model according to the vector they used even though the same antigen was used (148). To conclude, comparison of immune responses between survivors and vaccinees are crucial to improve the existing vaccines and develop the next-generation vaccines. One major evolution might be to design more targeted-vaccines using some specific EBOV GP and NP epitopes shown to be very immunogenic and to develop efficient vaccines against several Ebolavirus species.

#### Other Candidate Vaccines

Even though vaccines which were licensed or moved to late-stage clinical trials are based on viral vectors, other vaccine strategies have been investigated. Work using C57B6 or BALB/c mice vaccinated with EBOV Virus-like Particles (VLP) composed of GP and VP40, showed that mice survived primary challenge with MA-EBOV and that only if you adoptively transferred both serum and splenocytes into naïve mice would it rescue the mouse from MA-EBOV challenge. This confirms a role for both the humoral and cellular response in preventing fatal EVD following VLP vaccination (149). Furthermore, the same VLP were shown to protect NHPs against challenge with EBOV (150). DNA vaccination was also shown to induce EBOV-specific protective immune responses in Guinea pigs. In this case, immunisation with plasmids encoding for viral proteins such as EBOV GP, sGP, and NP generated antibody and T cell responses. Protection correlated with antibody titres and T cell responses to sGP or GP (151). Another study demonstrated that DNA vaccination boosted with recombinant adenoviral vectors encoding Ebola viral proteins could protect cynomolgus macaques against a challenge with a lethal dose of 1976 Mayinga strain of Zaire EBOV. Animals which were not vaccinated progressed to a moribund state and death in less than one week, while vaccinated animals were asymptomatic following this challenge for more than six months. The virus was not detected after the challenge and protection correlated with Ebola virus-specific CD8^+^ T cell and antibody responses (152, 153). In 2006, a three-plasmid DNA vaccine encoding GP and NP from EBOV as well as GP from SUDV was evaluated in a Phase I clinical trial in the US. This vaccine was well-tolerated and induced specific antibody responses to at least one of three antigens, especially GP from EBOV or SUDV, four weeks following the third vaccine dose. In addition, CD4^+^ T cell responses for GP (EBOV/SUDV) were detected in all vaccinees 2 weeks after the third vaccination (154). In 2011, Konduru et al. demonstrated that an EBOV GP fused to the Fc fragment of human IgG1 was able to generate neutralizing antibodies against rVSV-ZEBOV and T cell immunity against EBOV GP in mice. Here, seven/eight vaccinated mice were protected against challenge with MA-EBOV (111). Recently, a peptide vaccine based on a predominant NP epitope (NP44-52) found to induce CD8^+^ T cell response in EVD survivors was tested in mice. A single intradermal vaccination using an adjuvanted microsphere peptide vaccine formulation containing NP44-52 was shown to confer protective immunity against a MA-EBOV (155). Finally, mRNA vaccines based on EBOV GP and formulated with nanoparticles were demonstrated to induce GP-specific IgG and neutralizing antibodies in Guinea pigs. All vaccinated animals survived following an infection with a Guinea pig-adapted EBOV strain (156).

## Correlates of Protection

**Figure 3:**
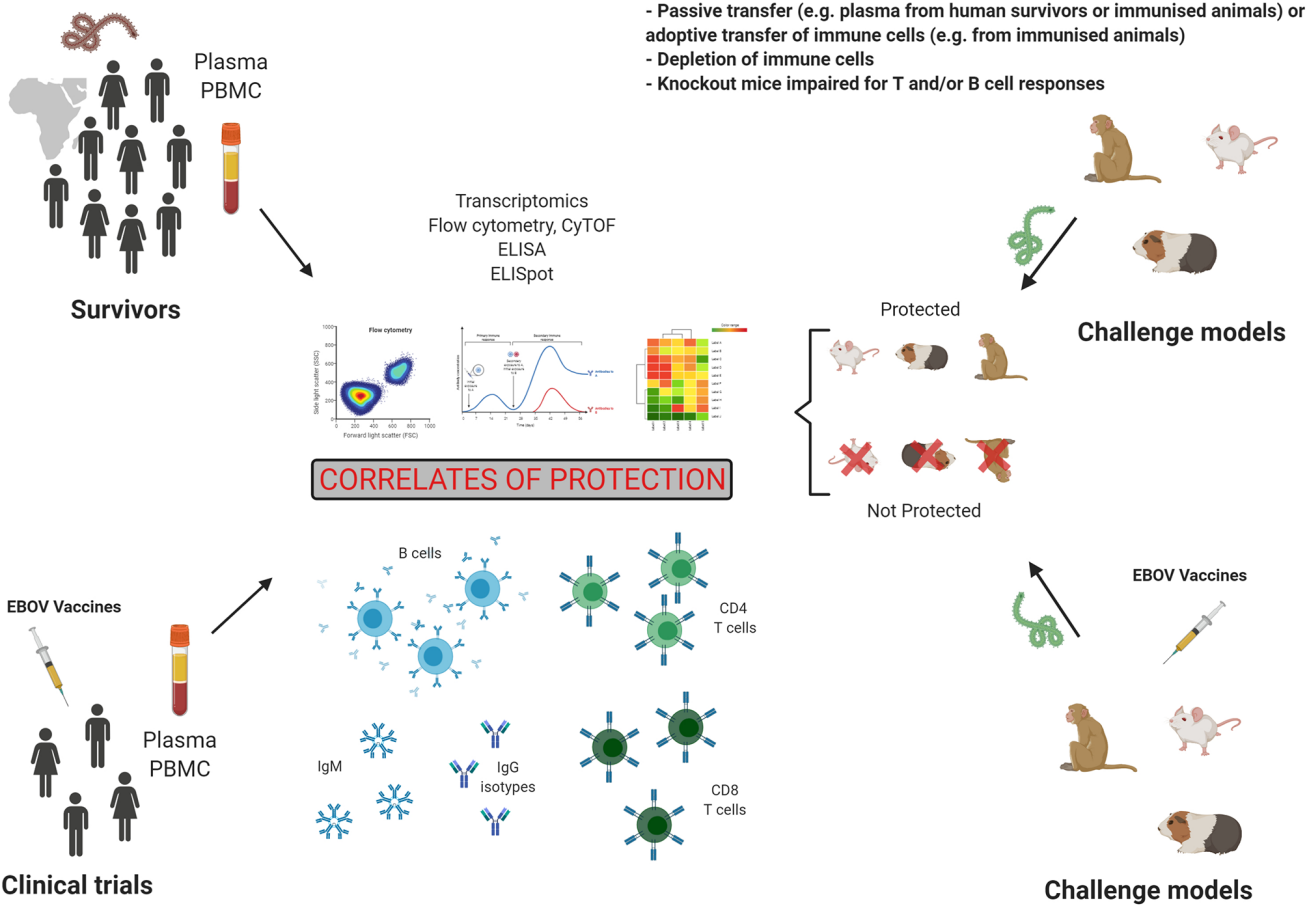
Preclinical and clinical studies in the search for correlates of protection. Studies of humoral and cellular responses in Evola virus disease (EVD) survivors and in vaccinated individuals is one strategy to establish correlates of protection. However, controlled challenge studies cannot be performed in humans. The second strategy to identify correlates of protection is the analysis of immune responses in challenge animal models, which allows to compare immunity in protected and not protected animals. Figure created with BioRender.

Immunology studies in survivors and animal models, as well as human clinical trials as shown in **Figure 3** have had a major impact on our understanding of EBOV pathogenesis, as well as therapeutics and vaccine development. These studies also informed on correlates of protection, which can be defined as an immune response that is responsible for protection. Following a natural infection, the antibody response, especially GP-specific neutralizing IgG in serum, is considered as a major correlate of protection in survivors and animal models, however it is also evident that those that succumb to infection will also have detectable levels of IgG in their serum. Therefore, it is not clear what titre is needed and whether antibodies to particular antigens/epitopes are needed to provide protection. It is likely that antibody as a correlate of protection will not only be defined as a quantifiable titre but also by the kinetics of such a response and that these combined factors should define antibody as a correlate of protection. In addition, the role of IgG Fc receptors in potential antibody-dependent enhancement of disease is not clear in EVD disease outcomes. The important role of frequency, activation, and phenotype of T cells in survival has been clearly demonstrated in humans and animal models. GP- but also NP-specific T cell responses have been shown to be involved in protection. However, the predominant role of either CD4^+^ or CD8^+^ T cells may vary according to animal models or human studies and like with the antibodies timing of these responses will be critical. Following vaccination with GP-based antigen, specific IgG responses are seen as the main correlate of protection in animal studies and clinical trials. Cellular mediated responses have been especially assessed in animal models. The frequency and activation of CD4^+^ and CD8^+^ T cells play a role in protection. However, the cytokine phenotype leading to protection varies between the studies and remains unclear. Finally, the role of innate immunity in survival is yet to be fully elucidated, and this will likely play a key role considering what is known about asymptomatic and mild disease and that these innate responses will inform on the adaptive ones. Therefore, protection may also correlate with innate immune signatures.

## Lessons Learned From the 2013–16 West Africa Epidemic

Interestingly, some similarities can be observed between the rapid EBOV research following the 2013–2016 West Africa epidemic and the current international research to control the COVID-19 pandemic. The last section discusses the lessons learned from the 2013–16 West Africa epidemic and the strategies used to tackle EBOV epidemics which could be applied for the COVID-19 pandemic.

A novel acute respiratory syndrome, now called Coronavirus disease-19 (COVID-19), was first identified in Wuhan (China) in December 2019. The genetic sequence of the causative agent was found to have similarity with two highly pathogenic respiratory Betacoronaviruses, SARS-CoV (157) and MERS-CoV (158) and was called SARS-CoV-2 (159). This virus has currently infected more than 71 million individuals resulting in >1.5 million deaths based on Johns Hopkins University’s live platform (160). Among the clinical signs of SARS-CoV-2 infection in humans, pneumonia was described in Chinese patients at the beginning of epidemic (161) (162). Later, a wider range of symptoms related to COVID-19 were described from mild-to-moderate symptoms including fever, cough, myalgia or loss of taste or smell to severe acute respiratory distress syndrome and sometimes multiorgan involvement, as well as shock. In severe cases, a cytokine storm (163) was shown to lead to systemic inflammatory response and endothelial damage, which may result in venous and arterial thrombotic events (164). The severity of disease was shown to be linked to advanced age and underlying conditions including hypertension, diabetes, cardiovascular disease, chronic respiratory disease and cancer (165). Even though information is still limited, post-COVID-19 symptoms have been observed in a significant number of patients at least for 4–8 weeks post-discharge from hospital (166). Interestingly, it was reported that some individuals were RT-PCR positive but were either asymptomatic or minimally symptomatic. Increasing evidences are showing that asymptomatic individuals can efficiently spread the virus (167).

To effectively tackle the COVID-19 ongoing pandemic, it is essential to come back to lessons learned from previous EVD epidemics, as well as to understand the similarities and differences between EVD epidemics and COVID-19 pandemic. It is crucial to evaluate the potential of strategies used to tackle EVD epidemics, especially the 2013–2016 West Africa epidemic, and to see if similar strategies could be applied for the current COVID-19 pandemic.

As in the context of EVD, the development of animal models that replicate human disease is a crucial step to study pathogenesis, establish correlates of protection, as well as assess the safety and efficacy of candidate vaccines and therapeutics. Some transgenic mouse (168–172), ferret (173–175), and NHP (176–178) models have been already developed to understand SARS-CoV-2 transmission, infection, as well as the development of local and systemic disease. Even though some asymptomatic cases have been suggested for EVD (70), the range of COVID-19 diseases seems to be wider from asymptomatic people (179) to mild-to-moderate disease in major cases and severe diseases in some individuals sometimes leading to death. Consequently, it becomes obvious that different types of animal models are necessary to replicate the range of illness severity and the variability of symptoms observed in humans. Animal models associated with studies in COVID-19 convalescents have a crucial role in dissection of protective immune responses and searching for correlates of protection.

During 2013–2016 and 2018 EVD epidemics in West Africa and DRC, clinical trials have been conducted in Ebola affected countries. Convalescent plasma (180, 181), monoclonal antibody (182, 183) and antiviral (184) therapies have been evaluated in the field. In the COVID-19 pandemic, the compassionate access to treatments and the implementation of clinical trials were rapidly adopted. Some drugs like hydroxychloroquine, remdesivir, lopinavir/ritonavir ± interferon beta-1A have been or are currently being evaluated in clinical trials including the Solidarity (185) and DisCoVeRy trials (**NCT04315948)**, while the Recovery trial is also testing the efficacy of anti-inflammatory drugs such as dexamethasone or Tocilizumab, the antibiotics Azythromycin, as well as COVID-19 convalescent plasma (186). In addition, the lead Ebola vaccines such as rVSV-ZEBOV, Ad26.ZEBOV-MVA-BN-Filo, ChAd3-EBO-Z were also successfully evaluated in the field (187). Currently, 163 COVID-19 vaccines are in preclinical evaluation and 51 are in clinical trials including 6 in Phase III based on WHO communication on the 2^nd^ of December 2020 (188). These candidate vaccines include replicating or non-replicating viral vectored vaccines, DNA vaccines, mRNA vaccines, autologous dendritic cell-based vaccine, and inactive virus vaccines (189). Some of them are currently in clinical trials overseas where the viral circulation is high. For instance, the efficacy of ChAdOx1 nCoV-19 also using a chimpanzee adenovirus vector similarly to ChAd3-EBO-Z, is currently under evaluation in Phase III trials in diverse population cohorts in the UK but also in Brazil and South Africa (190). The safety and immunogenicity of this vaccine in a prime-boost regimen was shown to be safe in young and old adults (191) and a Phase III interim analysis indicated that the vaccine was 70.4% effective when combining data from two dosing regimens but up to 90% efficacy in one regimen (192). The mRNA-based vaccine candidates BNT162b2 and mRNA-1273 announced to be 95% (193) and 94.5% effective (194), respectively. The mRNA-based vaccine BNT162b2 was approved by the UK regulators for use on the 2^nd^ of December 2020 (195), by Health Canada on the 9^th^ of December 2020 (196) and the FDA granted emergency use authorisation on the 11^th^ of December 2020 (197). Consequently, the integration of treatment and vaccine clinical trials into epidemic response is considered as one of best strategies to tackle epidemics and learn about immune responses in humans.

As in the context of EVD epidemics, international efforts from public health groups, universities, vaccine developers, regulators and funders are the key to progress in understanding SARS-CoV-2 infections, search for correlates of protection in COVID-19 convalescents and animal models, as well as to develop efficient therapeutics and vaccines.

## Author Contributions

SL and TT wrote the manuscript and designed the figures. JM designed the tables and revised the manuscript. MC revised the manuscript. All authors contributed to the article and approved the submitted version.

## Funding

SL, TT, and MC are supported by the US Food and Drug Administration (grant number: HHSF223201510104C). JM is funded *via* PHE PhD studentship program.

## Conflict of Interest

The authors declare that the research was conducted in the absence of any commercial or financial relationships that could be construed as a potential conflict of interest.
